# Identification of reliable QTLs and designed QTL breeding for grain shape and milling quality in the reciprocal introgression lines in rice

**DOI:** 10.1186/s12870-023-04707-9

**Published:** 2024-01-09

**Authors:** Mwenda Emelin, Xianjin Qiu, Fangjun Fan, Md. Alamin, Muhiuddin Faruquee, Hui Hu, Junying Xu, Jie Yang, Haiming Xu, Jauhar Ali, Bailong Liu, Yumin Shi, Zhikang Li, Luyan Zhang, Tianqing Zheng, Jianlong Xu

**Affiliations:** 1 Institute of Crop Sciences/State Key Laboratory of Crop Gene Resources and Breeding/National Key Facility for Crop Gene Resources and Genetic Improvement, Chinese Academy of Agricultural Sciences, Beijing, 100081 China; 2 Ministry of Agriculture and Rural Affairs (MARA) Key Laboratory of Sustainable Crop Production in the Middle Reaches of the Yangtze River (Co-Construction By Ministry and Province), College of Agriculture, Yangtze University, Jingzhou, 434025 China; 3 Institute of Food Crops, Jiangsu Academy of Agricultural Sciences, Jiangsu High Quality Rice Research and Development Center, Nanjing Branch of China National Center for Rice Improvement, Nanjing, 210014 China; 4 Institute of Crop Science and Institute of Bioinformatics, College of Agriculture and Biotechnology, Zhejiang University, Hangzhou, 310058 China; 5 International Rice Research Institute, Bangladesh Office, Dhaka, 1213 Bangladesh; 6 International Rice Research Institute, DAPO Box 7777, 1301 Metro Manila, Philippines; 7 Rice Research Institute, Guangxi Academy of Agricultural Sciences, Nanning, 530007 China; 8 National Nanfan Research InstituteChinese Academy of Agricultural Sciences, Sanya, 572024 China

**Keywords:** Reciprocal  introgression lines, Milling quality, Trait correlation, Genetic overlap, Multi-environment

## Abstract

Milling quality (MQ) and grain shape (GS) of rice (*Oryza sativa* L.) are correlated traits, both determine farmers’ final profit. More than one population under multiple environments may provide valuable information for breeding selection on these MQ-GS correlations. However, suitable analytical methods for reciprocal introgression lines with linkage map for this kind of correlation remains unclear. In this study, our major tasks were (1) to provide a set of reciprocal introgression lines (composed of two BC_2_RIL populations) suitable for mapping by linkage mapping using markers/bins with physical positions; (2) to test the mapping effects of different methods by using MQ-GS correlation dissection as sample case; (3) to perform genetic and breeding simulation on pyramiding favorite alleles of QTLs for representative MQ-GS traits. Finally, with four analysis methods and data collected under five environments, we identified about 28.4 loci on average for MQ-GS traits. Notably, 52.3% of these loci were commonly detected by different methods and eight loci were novel. There were also nine regions harboring loci for different MQ-GS traits which may be underlying the MQ-GS correlations. Background independent (BI) loci were also found for each MQ and GS trait. All these information may provide useful resources for rice molecular breeding.

**Supplementary Information:**

The online version contains supplementary material available at 10.1186/s12870-023-04707-9.

## Introduction

Rice (*Oryza sativa* L.) is a primary food crop, feeding nearly half of the world’s population. Profit for rice farmers largely depends on not only the grain yield but also the head rice rate during grain milling [1, 2]. Grain shape and milling quality are key determinants of the head rice rate. Grain shape is often characterized by grain length (GL) and grain width (GW), which are all typical complex quantitative traits [3]. Milling quality is often characterized by three component traits, namely brown rice rate (BR), milled rice rate (MR) and head rice rate (HR), which are also typical complex quantitative traits.

Compared to piles of works on grain shape, genetic studies focusing on milling quality in rice are still lagging behind. Initial results are often based on single-population under single environment [4, 5]. Later, populations derived from *Oryza glaberrima* [6] and *rufipogon* [7] were also adopted. Recently, works for GWAS by sequenced germplasm [8, 9] as well as QTL validation work by fine mapping [10] also provided valuable results. For complex traits, multiple populations and/or multiple environments data may be largely beneficial to genetic dissection. Genetic control of milling quality and grain shape was identified with single population under multiple seasons [11, 12] and with two RILs populations under two environments [13]. Data of two different populations were evaluated on QTL for milling quality and other traits [14].

Trait correlations or genetic overlaps are important especially for breeding selection. Recently, correlations between milling quality and grain yield were promoted [15]. However, grain yield and milling quality are both post-harvest traits. Comparatively, grain shape would be an index more suitable for field selection before harvest. Correlations between milling quality and grain shape traits were previously noted by phenotypic correlation with cultivars [16]. Notably, QTL mapping with single set of introgression lines has provided some different views [17–19]. Genetic studies focused on the relationship between these two traits especially with multiple populations under multiple environments, which may provide more information for utilization of QTLs for the genetic improvement of two groups of traits.

Meanwhile, consideration of a large number of QTLs is another challenge for breeders. Many factors limited the applications of detected QTLs, such as QTL by environment interactions and stability of QTL, different genetic backgrounds among populations, the linkage between detected QTL, false positives and false negatives, and lack of appropriate tools [20]. Breeding simulation study for target genotype provides a potential way to find the most appropriate crossing and pyramid desired alleles at various loci based on dependable QTL mapping results [20, 21].

In this study, we focused on QTL mapping of milling quality and grain shape using a set of reciprocal introgression lines developed by advanced backcrossing (BC_2_RIL) and a set of recombinant inbred lines (RIL) from a same cross. Our major tasks were (1) to construct a linkage map using a RIL population whose parents were the same as the two BC_2_RIL populations; (2) to conduct QTL mapping of the five traits in the two BC_2_RIL populations based on the re-constructed linkage map. Four different mapping methods were also used for comparison of detected QTLs; (3) to perform genetic and breeding simulation on pyramiding favorite alleles of QTLs for two representative traits, GL and HR.

## Materials and methods

### Genetic materials and genotyping

In our previous study, the development of a set of reciprocal introgression lines (BC_2_RILs) derived from Minghui 63 (MH63) and 02428 were described [22–24]. The BC_2_RILs consist of 424 lines, including 226 in MH63 background (MH63-ILs) and 198 in 02428 background (02428-ILs). All of them were genotyped by resequencing, and were used to construct a high-resolution map of 4568 bins. A set of RIL population consisting of 245 lines derived from the same parents was also adopted for linkage map construction.

### Field experiments and trait measurement

All 424 lines and their parents, MH63 and 02428, were planted in five environments in the south of China, including Shenzhen (SZ, 22.33°N, 114.07°E), Nanjing (NJ, NJ, 32.03°N, 118.46°E), Xuzhou (XZ, 34.15°N, 117.11°E), Jingzhou (JZ, 30.18°N, 112.15°E) and Sanya (SY, 18.31°N, 108.56°E).

Field experiments were conducted using a randomized complete design and followed the normal cultivating arrangement in each environment. Reciprocal ILs and their parents were planted in a three-row plot with ten individuals in each row with two replications. The planting density was 20 cm between rows and 20 cm between plants in each row. All field management followed local farmers’ practices. At maturity, eight individuals in the middle of each plot were harvested and bulked. After harvest, seeds were naturally dried and stored at room temperature for at least three months.

Milling quality traits were evaluated according to the National Rice Gain Quality Assessment Standard of China (GB/T17891-1999). For BR, about 30 g well-filled grains from each IL were weighed and then dehulled with an experimental dehuller (Taizhou Food and Oil Machinery Factory, JLGJ-45, Taizhou, China). Then the brown rice was weighed. The BR was expressed as (total brown rice weight / total paddy rice weight) × 100%. For MR, about 20 g brown rice was weighed and milled for 60 s with an experimental miller (Taizhou Food and Oil Machinery Factory, JMNJ-3, Taizhou, China) until the whiteness of the sample reached the China national standard of GB1354-2018 grade-I milled rice. MR was defined as (total milled rice weight / total brown rice weight) × BR × 100%. Similarly, HR was defined as (total head rice weight / total milled rice weight) × MR × 100%. All lines were measured twice, and the average value was adopted as trait values for mapping.

### Phenotypic data analysis

Analysis of variance (ANOVA) for phenotypic data and correlation analysis among traits were performed using the AOV function in QTL IciMapping V4.2 [25]. Heritability in broad sense on the genotypic mean level in each BC_2_RIL population was calculated for each environment and across environments by the two equations, respectively.

$$H_P^2\;=\;\frac{\sigma_G^2}{\sigma_P^2}\;=\;\frac{\sigma_G^2}{\sigma_G^2\;+\;{\displaystyle\frac1r}\;\sigma_\varepsilon^2}\;\mathrm{for}\;\mathrm{each}\;\mathrm{environment},\;\mathrm{and}$$

$$H_P^2=\;\frac{\sigma_G^2}{\sigma_P^2}\;=\;\frac{\sigma_G^2}{\sigma_G^2\;+{\displaystyle\frac1e}\sigma_{GE}^2+\;{\displaystyle\frac1{er}}\sigma_\varepsilon^2}\;\mathrm{across}\;\mathrm{environments}.$$

Best linear unbiased prediction (BLUP) values across multiple environments were calculated for each trait using the following model.

$${y}_{ijkl}=\mu +{g}_{i}+{e}_{j}+r/{e}_{k\left(j\right)}+ {ge}_{ij}+{\varepsilon }_{ijkl}$$

where *y*_*ijk*_ was the observed phenotypic value of individual *l* of the genotype *i* in block *k* at environment *j*; μ was the mean value of the population; *g*_*i*_ was the random effect of the *i*^th^ genotype; *e*_*j*_ was the random effect of the *j*^th^ environment; *r*/*e*_*k*(*j*)_ was the random effect of the *k*^th^ block in the *j*^th^ environment; *ge*_*ij*_ was the random effect of the genotype by environment interaction; *ε*_*ijkl*_ was the random residual effects.

### Linkage map construction using the RIL population

It’s notable that in the process of the BC_2_RILs construction, the recurrent parents (RPs), i.e. MH63 and 02428 were adopted as controls for phenotypic selection. According to previous experiences in reciprocal introgression line population construction [26], populations from interspecific cross in *indica* background tend to be more variated than that in *japonica* background and not easy to stabilize. Also, the phenotypic selection based on comparing to RP will largely affect the donor allelic frequency. Thus, we picked more lines with similar plant type and flowering time as MH63 during the selection of MH63-ILs, but selected more 02428-ILs largely varied from 02428 in agronomic traits except for the flowering time. Since this kind of artificial selection was conducted during the development of both BC_2_RIL populations, the RIL population was used for linkage map construction. Chromosome number and order of markers were anchored according to the physical map. MAP function in QTL IciMapping was adopted for estimating the genetic distance between markers. Recombination frequency was converted into map distance by the Kosambi mapping function. R package LinkageMapView was used for visualization of the linkage map [27].

### QTL mapping for grain shape and milling quality traits in the two BC_2_RIL populations

The algorithm of inclusive composite interval mapping (ICIM) for the BC_2_RIL population implemented by the BIP function in QTL IciMapping [28] was used for QTL mapping of the five traits in different environments, i.e. GL, GW, BR, MR and HR. QTL mapping was conducted in each environment separately, as well as the BLUP values across environments. The REG method proposed by Alamin et al. [29] was also adopted for QTL mapping in each environment as a comparison, which only output position and LOD score of each scanning position. As the two populations can also be viewed as CSSLs, we also used the RSTEP-LRT-ADD method in the CSL function of QTL IciMapping [30], which conducted QTL mapping in each environment separately as well as the means across environments. ICIM algorithm in MET function was adopted for QTL by [31].

The LOD threshold was set at 2.5 for all the first three methods, and at 4 for MET as all environments were analyzed at the same time in MET. The scanning step was 0.5 cM. The two probabilities for entering and removing variables were set at 0.001 and 0.002, respectively. Comparison of QTL mapping results among different environments, among the four mapping methods, and among the five traits was then conducted. QTLs in different populations were considered to be common if the genetic positions were close enough. In other words, distance in linkage map was less than 20 cM in terms of QTL positions. In individual populations, QTLs were considered to be stable if they were identified in at least two the environments for at least one population. Twenty cM was also adopted as the minimum distance to identify pleiotropy QTLs among traits. Locus detected throughout two populations (genetic backgrounds) was important and regarded as background independent locus (BI locus). A tool named shinyCircos was used for the visualization of QTL positions on the linkage map [32].

### Breeding simulation on pyramiding favorite alleles

Blib is a simulation platform for modelling, simulating, and predicting the genetic and breeding processes of different diploid species [33]. Our target of breeding design was to improve HR and GL. QTL for the two traits detected by all the four methods were considered, and those QTLs exhibiting consistent effect directions across environments were kept. For pleiotropy QTLs, if their effects for the two traits were opposite, HR was adopted as the primary index trait for determining the target genotype. The individuals mostly close to the target genotype were selected as parents for developing simulated progenies. Three types of crosses were considered, i.e. single cross, top cross and double cross. For each cross, 1000 DH lines were generated by Blib, and proportion of target genotypes in these lines was counted.

## Results

### Phenotypic evaluation

The descriptive statistics of grain shape and milling quality traits, i.e., GL, GW, BR, MR and HR of the two BC_2_RIL populations in different environments were shown in Table S1. The average values of the two parents, i.e., MH63 and 02428, in each environment were from 9.12 to 10.02 and 6.38 to 7.27 cm for GL, 2.59 to 2.98 and 3.30 to 3.56 cm for GW, 75.02 to 79.91 and 73.13 to 80.88% for BR, 64.06 to 69.01 and 57.68 to 68.48% for MR, 36.94 to 57.94 and 29.59 to 64.21% for HR. The average values of progenies in populations MH63-ILs and 02428-ILs in each environment were from 6.71 to 9.93 and 6.86 to 7.84 cm for GL, 2.41 to 3.02 and 3.20 to 3.48 cm for GW, 72.06 to 78.90% and 75.83 to 79.85% for BR, 62.01 to 72.62 and 61.27 to 67.67% for MR, 35.41 to 54.41 and 47.55 to 60.35% for HR. Obvious variations were observed in phenotypic data of parents and progenies in both populations, and so was the heritability among different environments (Table S1).

Table 1 shows the variance components and heritability in broad sense of the five traits across environments. Heritability of GL and GW was considerably high, i.e., over 0.90, and heritability of BR, MR and HR was a little lower, i.e., around 0.50 to 0.71. Pearson’s correlation coefficient between the traits across environments is shown in Table 2. A significant test indicated that GL was negatively correlated with GW, and BR, MR and HR were positively correlated in both populations. HR was negatively correlated with GL, and BR was positively correlated with GW. Other correlations were insignificant or inconsistent in the two genetic backgrounds.

**Table 1:** Variance components and heritability of the five traits in the two populations

**Trait**	**Population**	**Variance components**				**Heritability**^**a**^
		**Genotype**	**Environment**	**G by E Interaction**	**Random error**	
GL	MR63	0.16	1.99	0.05	0.03	0.95
	02428	0.35	0.13	0.03	0.04	0.97
GW	MR63	0.01	0.07	0.00	0.01	0.91
	02428	0.03	0.13	0.00	0.01	0.95
BR	MR63	1.34	5.15	2.14	10.45	0.55
	02428	1.40	2.53	2.32	8.69	0.50
MR	MR63	1.83	11.95	2.87	9.25	0.62
	02428	2.24	8.35	4.13	8.91	0.55
HR	MR63	12.38	61.64	21.38	26.28	0.71
	02428	26.58	28.95	108.16	26.85	0.52

*GL* grain length, *GW* grain width, *BR* brown rice rate, *MR* milled rice rate, *HR* head rice rate

^a^Heritability in broad sense

**Table 2:** Pearson’s correlation coefficient between the five traits across environments in the two populations

**Population**	**Trait**	**GL**	**GW**	**BR**	**MR**	**HR**
MR63	GL	1.0000				
	GW	-0.2116**	1.0000			
	BR	-0.0433	0.2719***	1.0000		
	MR	-0.1049	0.1268	0.8783***	1.0000	
	HR	-0.1935**	-0.1468*	0.3942***	0.5313***	1.0000
02408	GL	1.0000				
	GW	-0.3466***	1.0000			
	BR	-0.0650	0.2279**	1.0000		
	MR	0.0668	-0.1220	0.6301***	1.0000	
	HR	-0.2212**	0.0326	0.2566***	0.2861***	1.0000

*GL* grain length, *GW* grain width, *BR* brown rice rate, *MR* milled rice rate, *HR* head rice rate

Significant differences were indicated at ** for *P* = 0.01, and *** for *P* = 0.001

### Linkage map constructed in the RIL population

**Fig. 1:**
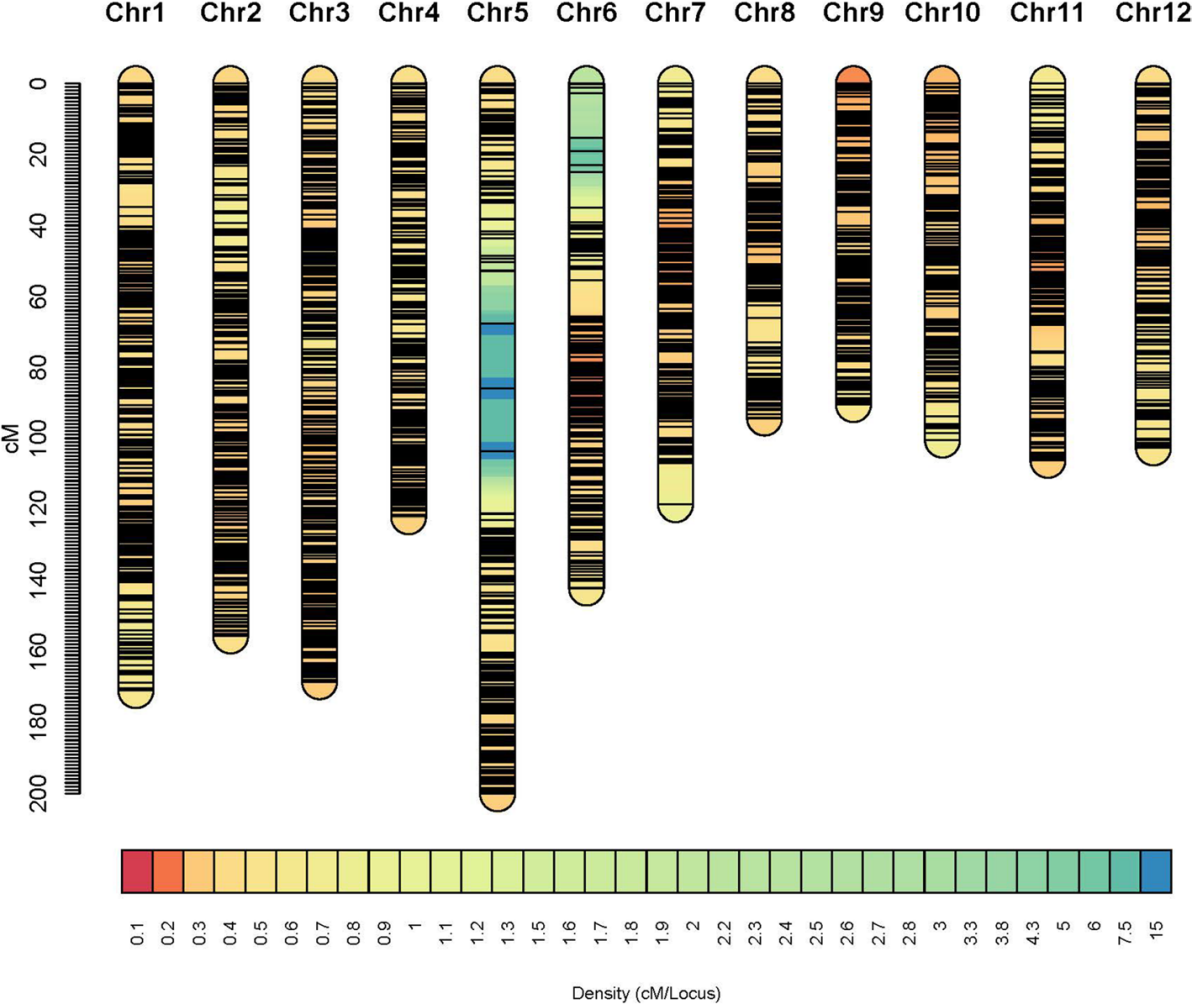
Linkage map constructed from the RIL population

The constructed linkage map is shown in Fig. 1, and general information on the linkage map is provided in Table 3. The whole genome spanned 1572.31 cM in length, consisting of 12 chromosomes. The number of markers was 4833 and the number of unique map positions (denoted as bin) was 2448. Chromosome 5 was the longest one with the length of 200.12 cM, while chromosome 9 was the shortest with the length at 90.50 cM. The largest gap was also observed on chromosome 5 with the length at 18.44 cM. Chromosome 3 had the largest number of markers, i.e., 319 markers, and chromosome 8 had the smallest number of markers, i.e. 153 markers. The average distance between markers was 0.33 cM in the whole genome, and the average distance between bins was 0.65 cM.

**Table 3:** Information on the linkage map constructed from the RIL population

**Chr**	**No. of markers**	**No. of bins**	**Length (cM)**	**Max distance (cM)**	**Average distance between markers (cM)**	**Average distance between bins (cM)**
1	504	291	171.01	6.66	0.34	0.59
2	477	255	155.64	3.78	0.33	0.61
3	596	319	168.54	3.10	0.28	0.53
4	336	204	121.99	2.71	0.36	0.60
5	319	201	200.12	18.44	0.63	1.00
6	394	168	142.11	12.53	0.36	0.85
7	428	192	118.62	11.70	0.28	0.62
8	308	153	94.34	6.84	0.31	0.62
9	370	164	90.50	4.06	0.25	0.56
10	371	158	100.51	4.15	0.27	0.64
11	392	183	106.24	7.41	0.27	0.58
12	338	160	102.70	3.56	0.30	0.65
Total	4833	2448	1572.31	18.44	0.33	0.65

### QTL detected by BIP

A total of 40, 28, 28, 21, and 22 QTLs were detected by BIP for GL, GW, BR, MR and HR, respectively (Table S2). QTLs detected in at least two environments for at least one population, were given in Table 4. For GL, 30 QTLs were stable and seven were detected in two genetic backgrounds (background independent, BI), i.e. *qGL.BIP-2.1*, *qGL.BIP-3.4*, *qGL.BIP-3.5*, *qGL.BIP-3.6*, *qGL.BIP-4.4*, *qGL.BIP-4.5* and *qGL.BIP-10.2*. For GW, 21 QTLs were stable, two of which were BI loci, i.e. *qGW.BIP-1.2* and *qGW.BIP-5.1*. For BR, 13 QTLs were stable, and no BI locus was found. For MR, 11 QTLs were stable, one was BI locus, i.e. *qMR.BIP-2.2*. For HR, 9 QTLs were stable, one BI locus, i.e. *qHR.BIP-2.1* was found. Most stable QTLs had the same additive effect directions across populations and environments. For example, the additive effect of *qGL.BIP-2.1* ranged from 0.09 to 0.16 in MH63-ILs, and from 0.14 to 0.18 in 02428-ILs. Some exceptions were also observed, for example, *qHR.BIP-7.1* was stable in MH63-ILs, whose additive effect ranged from -2.51 to 2.02.

**Table 4:** Stable QTLs detected by BIP

***Loci***	**Pop.**^**a**^	**Env.**^**b**^	**Pos. (cM)**^**c**^	**LOD**	**PVE (%)**^**d**^	**Add.**^**e**^
*qGL.BIP-1.1*	MR63	SY/SZ	8.5–14	4.75–9.21	1.42–3.39	-0.14 to -0.11
*qGL.BIP-1.2*	MR63	BJ/JZ/SY/SZ/B	41.5–67	3.39–52.92	2.32–16.33	-0.08 to 0.32
*qGL.BIP-1.3*	MR63	HN/NJ/XZ	108.5–110.5	4.48–7.57	0.76–2.89	0.44 to 0.92
*qGL.BIP-2.1*	MR63	BJ/SY/B	6–12.5	6.23–10.60	1.73–5.95	0.09 to 0.16
	02428	JZ/NJ	16.5	3.92–4.77	4.28–4.34	0.14 to 0.18
*qGL.BIP-2.2*	02428	NJ/SY	70	3.50–4.29	4.70–4.84	-0.12 to -0.11
*qGL.BIP-2.3*	MR63	HN/JZ/SZ/B	91.5–112.5	2.82–11.16	1.33–4.54	-0.24 to 0.33
*qGL.BIP-2.4*	MR63	JZ/B	129.5–130	6.11–13.96	2.77–3.30	-0.17 to -0.14
*qGL.BIP-3.1*	MR63	HN/XZ/B	5.5–7.5	3.33–8.23	1.16–4.71	0.22 to 0.29
*qGL.BIP-3.2*	MR63	BJ/SY	30–34	4.16–5.96	1.47–4.92	0.18 to 0.29
*qGL.BIP-3.4*	MR63	BJ/HN/JZ/NJ/SY/SZ/XZ/B	92–102	10.13–51.15	2.09–31.28	0.32 to 0.64
	02428	JZ/NJ/SZ/B	93–94.5	7.10–53.05	13.21–43.96	0.23 to 0.80
*qGL.BIP-3.5*	MR63	NJ/SY/SZ/XZ	116–118	5.10–58.85	1.52–19.13	-1.06 to 0.34
	02428	SY/SZ/SZ/B	115	5.60–7.99	2.99–16.83	0.19 to 0.28
*qGL.BIP-3.6*	MR63	NJ/SZ/B	130–130.5	31.24–83.48	7.49–31.06	0.27 to 1.38
	02428	JZ/NJ/SY/SZ/B	137–149.5	3.33–9.25	1.66–9.11	0.13 to 0.30
*qGL.BIP-4.4*	MR63	JZ/SZ	83.5–84	4.06–35.63	1.94–14.62	0.26 to 0.73
	02428	JZ/SZ/XZ/B	86–87	6.11–9.31	3.77–17.09	0.17 to 0.26
*qGL.BIP-4.5*	MR63	HN/SY/B	102.5–105.5	4.11–7.87	1.21–2.79	0.09 to 0.14
	02428	NJ/SY	112	3.70–5.42	5.40–6.67	0.14 to 0.17
*qGL.BIP-5.1*	MR63	SY/SZ/B	24.5–25	6.43–8.84	1.18–3.10	0.09 to 0.15
*qGL.BIP-5.4*	02428	SZ/XZ/B	173.5	3.41–5.98	1.77–8.93	0.16 to 0.18
*qGL.BIP-5.5*	02428	NJ/SY	194	3.20–4.54	4.87–7.82	0.19 to 0.21
*qGL.BIP-6.1*	MR63	HN/XZ	25.5–26	4.86–6.99	1.06–3.29	0.67 to 0.92
*qGL.BIP-7.1*	MR63	HN/NJ/SY/XZ/B	29–48	4.06–65.30	1.40–13.33	0.13 to 0.83
*qGL.BIP-7.2*	MR63	JZ/NJ/SZ	67–68	16.94–22.16	2.52–9.47	0.23 to 0.31
*qGL.BIP-7.3*	MR63	HN/NJ/SZ	97.5–103.5	4.39–5.88	0.63–3.96	0.11 to 0.53
*qGL.BIP-8.1*	MR63	HN/SY/SZ/XZ/B	6.5–23	2.64–13.59	1.51–2.93	0.11 to 0.47
*qGL.BIP-8.2*	MR63	BJ/NJ/SY/SZ	62.5–82	6.89–13.39	0.99–11.93	-0.25 to -0.11
*qGL.BIP-9*	MR63	BJ/HN	38	3.61–6.40	3.96–4.19	0.29 to 0.53
*qGL.BIP-10.1*	MR63	NJ/JZ/SY/SZ/XZ	34.5–46.5	3.83–7.54	0.39–2.79	-0.23 to -0.13
*qGL.BIP-10.2*	MR63	HN/JZ/SY/SZ/XZ/B	60.5–66.5	2.51–28.59	1.06–8.58	0.14 to 0.92
	02428	NJ/SY/SZ/XZ/B	73	3.63–8.43	1.79–12.26	0.16 to 0.23
*qGL.BIP-11.1*	MR63	BJ/HN/NJ/SZ/XZ	12–21.5	5.01–25.98	1.86–9.85	0.09 to 0.35
*qGL.BIP-11.2*	MR63	NJ/XZ	40	2.89–3.16	0.36–0.44	-0.18 to -0.17
*qGL.BIP-12.1*	MR63	NJ/SZ	38–45	3.16–5.81	0.63–0.95	0.17 to 0.29
*qGL.BIP-12.2*	MR63	HN/XZ	78.5–83.5	3.77–5.65	1.06–3.28	0.68 to 0.92
*qGW.BIP-1.2*	MR63	BJ/HN/JZ/NJ/SZ/SY/XZ/B	42–60.5	2.60–26.53	0.52–10.11	0.03 to 0.11
	02428	SZ/B	67–72.5	3.57–6.44	4.58–7.11	-0.10 to -0.07
*qGW.BIP-1.4*	MR63	HN/JZ/NJ/SY/B	157.5–171	3.85–10.80	1.14–3.34	-0.04 to -0.03
*qGW.BIP-2.1*	MR63	SY/SZ	54.5–67.5	12.34–16.79	3.77–5.80	-0.08 to 0.25
*qGW.BIP-2.2*	MR63	SY/B	98.5–112.5	3.64–7.32	1.08–3.23	-0.07 to -0.02
*qGW.BIP-3.1*	02428	NJ/SZ/XZ/B	30.5	3.10–4.43	3.13–6.93	-0.07 to -0.04
*qGW.BIP-3.2*	MR63	BJ/HN/SY/SZ/XZ/B	147–168	7.51–24.41	1.64–9.66	-0.23 to -0.08
*qGW.BIP-4.1*	MR63	HN/SY/B	40–43.5	4.13–12.78	1.28–4.87	-0.07 to -0.05
*qGW.BIP-4.2*	MR63	SZ/XZ	81–84	4.40–41.75	0.92–12.55	-0.29 to 0.09
*qGW.BIP-4.3*	02428	NJ/SY/SZ/B	108.5	3.04–8.28	4.87–11.85	-0.08 to -0.05
*qGW.BIP-5.1*	MR63	BJ/HN/JZ/NJ/SY/SZ/XZ/B	28–31	9.67–92.26	4.18–51.4	-0.29 to -0.07
	02428	JZ/NJ/SY/SZ/XZ/B	28.5	9.3–25.67	17.63–36.47	-0.15 to -0.12
*qGW.BIP-5.2*	MR63	HN/NJ/XZ	66.5–73	2.60–5.49	1.53–2.76	-0.26 to -0.13
*qGW.BIP-5.3*	MR63	BJ/HN/XZ	105–112.5	2.55–5.58	1.56–6.75	-0.26 to 0.14
*qGW.BIP-5.5*	MR63	BJ/HN/NJ/SY	179.5–184	4.18–13.27	2.18–6.36	0.04 to 0.06
*qGW.BIP-9.1*	MR63	HN/NJ	8.5–26.5	5.89–11.92	1.81–5.22	0.07 to 0.12
*qGW.BIP-9.2*	MR63	JZ/SZ/B	86.5–89	3.09–6.94	1.40–2.19	-0.05 to -0.02
*qGW.BIP-10.2*	MR63	HN/XZ	61–72	3.90–34.91	2.39–9.87	-0.26 to -0.19
*qGW.BIP-11.1*	MR63	HN/NJ	9–11	3.54–5.77	1.42–2.39	-0.26 to 0.04
*qGW.BIP-12.1*	MR63	HN/SY	13–14.5	5.90–9.03	2.76–2.88	-0.08 to -0.05
*qGW.BIP-12.2*	MR63	JZ/NJ/B	33–38	3.74–9.57	2.01–3.74	0.05 to 0.10
*qGW.BIP-12.3*	02428	JZ/NJ/SY/SZ/XZ/B	81.5–89	6.00–9.75	8.81–16.35	-0.09 to -0.06
*qBR.BIP-1.4*	MR63	BJ/SY/SZ	97–108.5	2.50–3.87	0.83–1.82	3.11 to 6.15
*qBR.BIP-1.6*	MR63	BJ/SZ	168–168.5	3.30–4.18	1.29–2.42	2.33 to 6.97
*qBR.BIP-2.1*	02428	NJ/B	0–16.5	5.27–5.61	4.92–7.65	-0.22 to -0.74
*qBR.BIP-2.3*	MR63	JZ/XZ	104.5–113	3.99–11.48	0.31–2.05	-1.27 to 12.08
*qBR.BIP-2.4*	MR63	HN/B	135.5–146	3.04–7.54	4.27–6.33	0.66 to 1.85
*qBR.BIP-3.1*	MR63	HN/NJ/B	4.5–23	4.59–5.34	0.89–4.32	0.58 to 2.67
*qBR.BIP-3.3*	02428	NJ/B	114.5–130.5	3.67–14.65	5.89–14.27	-1.35 to 0.20
*qBR.BIP-5.1*	MR63	HN/B	30–30.5	2.71–6.41	2.24–5.04	-1.16 to -0.39
*qBR.BIP-5.2*	MR63	HN/JZ	74.5	2.86–22.06	0.37–7.55	3.46 to 10.00
*qBR.BIP-5.3*	MR63	HN/JZ	112	2.66–21.86	0.37–7.48	3.44 to 10.00
*qBR.BIP-5.4*	MR63	HN/B	184	6.40–17.94	5.40–15.96	0.52 to 1.35
*qBR.BIP-11.1*	MR63	JZ/NJ/SY/SZ	26–38.5	2.92–12.68	0.31–2.19	3.99 to 12.31
*qBR.BIP-12*	MR63	JZ/XZ	34–34.5	10.35–25.04	0.34–17.06	5.76 to 9.99
*qMR.BIP-1.2*	MR63	HN/B	140–140.5	2.98–5.64	2.66–6.12	0.29 to 0.90
*qMR.BIP-2.1*	02428	XZ/B	81	4.87–5.82	7.54–9.00	0.28 to 1.05
*qMR.BIP-2.2*	MR63	JZ/XZ	104.5–112.5	4.24–4.42	0.46–3.83	-1.42 to 9.45
	02428	XZ/B	104	4.49–6.05	9.01–10.48	-1.26 to -0.33
*qMR.BIP-2.3*	MR63	HN/B	130–148.5	6.13–7.44	5.67–8.21	0.38 to 1.59
*qMR.BIP-3.1*	MR63	HN/NJ	5–23	4.08–5.84	3.84–10.50	1.57 to 2.20
*qMR.BIP-3.2*	02428	NJ/XZ/B	113–130.5	3.83–5.30	6.63–7.53	-0.91 to 1.28
*qMR.BIP-5.1*	MR63	HN/JZ	73.5–75	2.68–19.01	0.59–7.13	3.39 to 8.53
*qMR.BIP-5.3*	MR63	BJ/HN/NJ/SY/SZ/XZ/B	184–194.5	2.98–19.15	5.84–23.87	0.61 to 1.70
*qMR.BIP-10*	MR63	HN/XZ	61	2.70–3.04	3.93–5.15	3.06 to 3.16
*qMR.BIP-11.1*	MR63	JZ/SZ	26–38	2.91–5.29	0.44–8.22	6.78 to 9.65
*qMR.BIP-12*	MR63	JZ/XZ/B	33.5–37.5	3.06–7.99	0.58–4.61	0.58 to 8.00
*qHR.BIP-1.1*	MR63	XZ/B	108.5–109	2.62–2.78	3.55–4.19	-5.91 to -1.17
*qHR.BIP-1.2*	MR63	BJ/NJ/XZ/B	161.5–164	3.00–4.91	0.74–4.96	0.58 to 2.26
*qHR.BIP-2.1*	MR63	NJ/XZ/B	98–102.5	2.99–4.37	2.71–3.00	-3.61 to -1.13
	02428	JZ/SZ	110.5–111.5	4.32–4.44	7.40–9.24	-3.19 to -2.75
*qHR.BIP-3.1*	MR63	JZ/SY	47–54	2.87–2.91	3.69–6.03	-4.16 to -3.82
*qHR.BIP-3.2*	MR63	SZ/B	93–98.5	3.84–6.92	5.61–7.01	-5.00 to -1.49
*qHR.BIP-4.1*	MR63	SY/XZ	17–32.5	5.22–7.92	3.25–10.75	3.22 to 5.48
*qHR.BIP-5.1*	MR63	NJ/SY/XZ/B	7.5–20	6.19–14.30	4.95–15.75	0.92 to 4.05
*qHR.BIP-7.1*	MR63	BJ/XZ	20.5–30	3.81–4.33	0.87–2.58	-2.51 to 2.02
*qHR.BIP-10.2*	MR63	BJ/XZ/B	86–93.5	3.65–26.46	2.16–27.36	2.50 to 4.65

*GL* grain length, *GW* grain width, *BR* brown rice rate, *MR* milled rice rate, *HR* head rice rate

^a^Population

^b^Environment

^c^Position

^d^Percentage of phenotypic variance explained

^e^Additive effect

B: Best linear unbiased prediction (BLUP)

### QTL detected by REG

A total of 35, 32, 42, 43, and 37 QTLs were detected by REG for GL, GW, BR, MR and HR, respectively, and the related information was listed in Table S3. QTLs detected in at least two environments for at least one population, were given in Table 5. For GL, 26 QTLs were stable, 7 of which were BI loci, i.e. *qGL.REG-2.1*, *qGL.REG-2.2*, *qGL.REG-3.2*, *qGL.REG-6.1*, *qGL.REG-7.1*, *qGL.REG-9.1* and *qGL.REG-12.1*. For GW, 8 of 11 stable QTLs were BI loci, i.e. *qGW.REG-1.2*, *qGW.REG-4.3*, *qGW.REG-5*, *qGW.REG-9.1*, *qGW.REG-9.2*, *qGW.REG-10.2*, *qGW.REG-11.2* and *qGW.REG-12.3*. For BR, 8 of 25 stable QTLs were BI loci, i.e. *qBR.REG-2.3*, *qBR.REG-3.3*, *qBR.REG-4.1*, *qBR.REG-5.3*, *qBR.REG-7.3*, *qBR.REG-9.2*, *qBR.REG-11.2* and *qBR.REG-12.3*. For MR, 12 of 22 stable QTLs were BI loci, i.e. *qMR.REG-1.3*, *qMR.REG-2.2*, *qMR.REG-2.4*, *qMR.REG-4.4*, *qMR.REG-5.4*, *qMR.REG-6.2*, *qMR.REG-7.1*, *qMR.REG-8.1*, *qMR.REG-8.2*, *qMR.REG-10.1*, *qMR.REG-11.1* and *qMR.REG-11.3*. For HR, 8 of 25 stable QTLs were BI loci, i.e. *qHR.REG-1.3*, *qHR.REG-2.1*, *qHR.REG-5.3*, *qHR.REG-6.1*, *qHR.REG-6.3*, *qHR.REG-7.1*, *qHR.REG-8.1* and *qHR.REG-11.3*. REG detected more QTLs and more stable QTLs than did BIP. Additive effects and PVE of QTLs were not provided by the REG method.

**Table 5:** Stable QTLs detected by REG

***Loci***	**Pop.**^**a**^	**Env.**^**b**^	**Pos. (cM)**^**c**^	**LOD**
*qGL.REG-1.3*	02428	NJ/JZ/XZ	114	16.5–21.8
*qGL.REG-1.4*	MR63	HN/JZ/NJ/SZ/XZ/B	128.45–143	28.8–368.0
*qGL.REG-1.5*	02428	SY/SZ/B	163.9	18.9–29.3
*qGL.REG-2.1*	MR63	BJ/HN/XZ	3.39–11	39.0–251.0
	02428	JZ/NJ	8.5–18.8	17.9–21.2
*qGL.REG-2.2*	MR63	NJ/SY/SZ/B	50.3–57	26.3–372
	02428	JZ/XZ	46–46.03	14.0–24.1
*qGL.REG-3.2*	MR63	BJ/JZ/NJ /SY/SZ/XZ/B	95–97.5	38.0–395.0
	02428	JZ/NJ/SZ/B	79.2–93.7	39.4–61.0
*qGL.REG-3.3*	02428	SY/XZ	114.9–123.3	35.2–35.6
*qGL.REG-4.1*	MR63	JZ/SY/SZ/XZ/B	70.41–86	26.1–51.2
*qGL.REG-4.2*	MR63	HN/JZ/NJ/SY/SZ/SZ/B	95.75–105.52	14.7–370.0
*qGL.REG-5.1*	02428	JZ/NJ/XZ/B	4.5–5.0	14.6–19.6
*qGL.REG-5.5*	MR63	JZ/NJ/SY/SZ/B	180–182.8	14.4–363.0
*qGL.REG-6.1*	MR63	JZ/NJ/SY/SZ/XZ/B	13.5–16	12.5–683.0
	02428	JZ/NJ/SY/SZ/B	22–23.5	16.3–34.9
*qGL.REG-6.2*	MR63	BJ/HN	130–139	23.8–28.0
*qGL.REG-7.1*	MR63	BJ/JZ/NJ/SY/SZ/XZ/B	28.5–46.39	24.7–371.0
	02428	JZ/SY/SZ/XZ/B	29.2–33.42	19.1–26.0
*qGL.REG-7.2*	MR63	BJ/HN	87.5–96.5	24.3–35.0
*qGL.REG-8.1*	MR63	BJ/JZ/NJ/SY/SZ/XZ/B	18.25–42.81	17.3–380.0
*qGL.REG-8.2*	02428	JZ/NJ/SY/SZ/XZ/B	90–93.21	14.2–24.1
*qGL.REG-9.1*	MR63	BJ/HN/JZ/NJ/SY/SZ/XZ/B	32–38	18.7–697.0
	02428	JZ/NJ	45.8	16.3–27.6
*qGL.REG-9.2*	02428	SY/SZ/XZ/B	73.38–73.4	28.1–34.5
*qGL.REG-10.1*	02428	JZ/NJ/SZ/XZ/B	39.5–40	22.9–35.9
*qGL.REG-10.2*	MR63	BJ/HN/JZ/NJ/SY/SZ/XZ/B	64.5–66.55	24.7–374.0
*qGL.REG-11.1*	MR63	BJ/HN/JZ/NJ/SY/SZ/XZ/B	1–12	24.1–390.0
*qGL.REG-11.2*	02428	JZ/NJ/SY/SZ/XZ/B	92.87–93	10.6–37.8
*qGL.REG-12.1*	MR63	JZ/SY	0–0.219	13.0–263.0
	02428	JZ/NJ/SY/SZ/XZ/B	12–34	14.7–26.2
*qGL.REG-12.2*	MR63	HN/NJ	45.5	15.1–30.9
*qGL.REG-12.3*	MR63	BJ/SZ/XZ/B	64.7–92.5	22.4–804.0
*qGW.REG-1.1*	MR63	HN/JZ/SZ/XZ	38.5–60.57	49.1–273.0
*qGW.REG-1.2*	MR63	BJ/NJ/SY	82.1–97	26.4–106.0
	02428	JZ/NJ/SZ/XZ/B	80.9–84.2	18.1–27.3
*qGW.REG-2.1*	02428	JZ/NJ/SY/SZ/XZ/B	8.5–18.8	25.25–47.5
*qGW.REG-2.2*	MR63	JZ/SZ/XZ	69.5–70.5	55.5–106.4
*qGW.REG-2.3*	MR63	BJ/HN/NJ/SY/B	95.5–98.3	25.6–276.0
*qGW.REG-3.1*	02428	JZ/NJ/SY/XZ/B	30–30.5	13.98–23.1
*qGW.REG-3.2*	MR63	BJ/HN/JZ/NJ/SY/SZ/XZ/B	141.7–167.5	37.7–280.0
*qGW.REG-4.1*	MR63	NJ/B	6.8–15.5	23.1–50.4
*qGW.REG-4.2*	MR63	HN/SY/SZ/XZ	31.5–42.5	98.7–265.0
*qGW.REG-4.3*	MR63	BJ/KZ	96.5–99.3	40.8–106.0
	02428	JZ/NJ/SY/SZ/XZ/B	108.8–111.8	26.0–46.0
*qGW.REG-5*	MR63	BJ/HN/JZ/NJ/SY/SZ/XZ/B	20.1–29.5	73.0–330.0
	02428	JZ/NJ/SY/SZ/XZ/B	28.7	48.42–82.46
*qGW.REG-6.1*	02428	JZ/NJ/SZ/B	2.5–6.5	18.7–24.2
*qGW.REG-6.2*	MR63	BJ/NJ/SY/SZ/XZ/B	18.5–37	33.1–241.0
*qGW.REG-6.3*	02428	SY/XZ	54.5–55.4	23.6–27.98
*qGW.REG-6.4*	MR63	HN/JZ	103.5	48.7–278.0
*qGW.REG-7.1*	02428	JZ/NJ/SY/SZ/XZ/B	26.7–39.4	13.46–29.7
*qGW.REG-7.2*	MR63	BJ/HN/JZ/NJ/SY/SZ/XZ/B	86.2–97.5	51.6–267.0
*qGW.REG-8.1*	MR63	NJ/SY/SZ/XZ/B	20.9–33.8	49.1–237.0
*qGW.REG-8.2*	02428	JZ/NJ/SY/SZ/XZ/B	57.4	15.75–30.5
*qGW.REG-8.3*	MR63	BJ/HN	91.5–93.21	109.0–269.0
*qGW.REG-9.1*	MR63	HN/NJ/XZ	5.78–27	18.5–278.0
	02428	SY/SZ/B	14.8	8.42–11.7
*qGW.REG-9.2*	MR63	BJ/JZ.SY/SZ	37.5–49.7	48.6–240.0
	02428	NJ/XZ	31.1–33.8	10.3–11.2
*qGW.REG-10.1*	MR63	BJ/HN/JZ/SY/SZ	2.23–39.9	52.7–271.0
*qGW.REG-10.2*	MR63	NJ/XZ/B	81.5–93.8	26.3–104.5
	02428	JZ/NJ/SY/SZ/XZ/B	81.5–98.6	18.8–34.9
*qGW.REG-11.1*	MR63	JZ/NJ/SZ/XZ	31.7–33	26.3–105.9
*qGW.REG-11.2*	MR63	BJ/HN/SY/B	60.23–65.7	54.7–263.0
	02428	JZ/NJ/SY/SZ/XZ/B	47.2–51.7	35.69–56.4
*qGW.REG-12.2*	MR63	BJ/NJ/SY/B	46–58	27.1–250.0
*qGW.REG-12.3*	MR63	JZ/SZ/XZ	78.5–95.5	56.1–104.5
	02428	JZ/NJ/SY/SZ/XZ/B	68.6–81.5	32.91–68.0
*qBR.REG-1.1*	MR63	NJ/SY/SZ/XZ	9.21–25.33	6.22–273.0
*qBR.REG-1.2*	02428	JZ/NJ/SZ/XZ	51.1–72	5.02–12.93
*qBR.REG-1.5*	MR63	BJ/B	160.49–171.01	10.01–201.0
*qBR.REG-2.1*	02428	NJ/SZ	0.907–1.5	5.88–8.24
*qBR.REG-2.2*	MR63	BJ/JZ/SZ	28.6–31.4	17.6–270.0
*qBR.REG-2.3*	MR63	SY/XZ/B	58.46–67.07	10.75–20.8
	02428	JZ/XZ/B	70.06–70.1	3.6–10.62
*qBR.REG-3.1*	MR63	JZ/NJ	14.1–23	16.3–272.0
*qBR.REG-3.3*	MR63	HN/SY	83–88.5	21.2–247.0
	02428	JZ/XZ	87–103.47	2.64–8.91
*qBR.REG-3.4*	MR63	BJ/XZ/B	135.63–145.03	16.52–200.0
*qBR.REG-3.5*	02428	NJ/SY/B	165.43–165.6	6.99–10.35
*qBR.REG-4.1*	MR63	BJ/SY/XZ/B	5–11.02	12.52–201.0
	02428	SY/SZ/XZ	6.5–24.5	5.34–6.69
*qBR.REG-4.4*	MR63	HN/NJ	96.5–106.27	248.0–259.0
*qBR.REG-5.3*	MR63	BJ/HN/SY/SZ/XZ/B	184.31–192.53	20.1–281.0
	02428	JZ/SY/SZ/XZ/B	169.12–200	2.62–6.74
*qBR.REG-6.1*	02428	NJ/B	20.5–21	3.27–6.09
*qBR.REG-6.2*	MR63	BJ/NJ	40.99–51.6	201.0–257.0
*qBR.REG-6.3*	MR63	JZ/SZ	72.3–83.2	16.7–270.0
*qBR.REG-6.4*	MR63	HN/SY/XZ/B	106.55–134.92	14.3–251.0
*qBR.REG-7.1*	02428	JZ/SY	5.06–14.51	6.26–7.19
*qBR.REG-7.3*	MR63	BJ/HN/JZ/NJ/SZ/XZ/B	84.4–98.5	5.44–260.0
	02428	SZ/B	83.06–86.5	4.05–6.87
*qBR.REG-8.1*	02428	JZ/SY/SZ/XZ/B	11.93–34.37	2.85–7.85
*qBR.REG-8.2*	MR63	JZ/XZ/B	44.34–46.5	10.51–21.21
*qBR.REG-8.3*	MR63	HN/NJ/SY/SZ	65–91.5	23.3–273.0
*qBR.REG-9.1*	MR63	HN/NJ/SZ/XZ/B	5.78–40.5	4.74–272.0
*qBR.REG-9.2*	MR63	BJ/JZ/SY	57.5–87.6	14.2–195.0
	02428	JZ/NJ/SY/XZ/B	57.5–77.5	3.57–10.67
*qBR.REG-10.1*	02428	JZ/SY/B	5.61–32.9	2.5–5.96
*qBR.REG-10.2*	MR63	HN/JZ/NJ/SY/SZ/XZ/B	72.3–97.1	16.3–271.0
*qBR.REG-11.1*	02428	SZ/XZ	3–7.5	4.96–5.95
*qBR.REG-11.2*	MR63	BJ/JZ/NJ/XZ/B	21.55–38	17.2–260.0
	02428	JZ/NJ/SY/B	41–51.68	3.33–8.12
*qBR.REG-11.3*	MR63	HN/SY	82.81–97.35	21.6–247.0
*qBR.REG-12.1*	MR63	SZ/B	11	6.78–273.0
*qBR.REG-12.2*	02428	XZ/NJ	33.4	4.49–4.53
*qBR.REG-12.3*	MR63	BJ/JZ/NJ/SY/XZ	78.8–94.16	9.78–259.0
	02428	SY/B	64–72.7	5.26–5.59
*qMR.REG-1.1*	02428	JZ/NJ/B	0–22.8	3.85–9.94
*qMR.REG-1.2*	MR63	JZ/NJ/SZ	62.3–84.2	3.29–10.79
*qMR.REG-1.3*	MR63	BJ/HN/XZ/B	122.4–156.5	9.32–12.64
	02428	SY/SZ/XZ	108.34–135.3	9.32–12.64
*qMR.REG-2.2*	MR63	BJ/SZ	69.5–85.74	4.05–4.73
	02428	SY/XZ/B	68.4–70.06	6.25–9.46
*qMR.REG-2.3*	MR63	NJ/SY/XZ	97.5–112	2.87–16.77
*qMR.REG-2.4*	MR63	HN/B	135.58–148.52	16.27–19.92
	02428	JZ/SZ	148.5	6.26–6.4
*qMR.REG-3.3*	MR63	BJ/HN/XZ	50.82–61.53	8.5–31.19
*qMR.REG-3.4*	02428	NJ/JZ/SZ/B	92.5–130.5	5.33–11.52
*qMR.REG-3.5*	MR63	JZ/SZ/B	146.1–164.9	3.16–21.43
*qMR.REG-4.1*	MR63	BJ/SY/SZ/B	2.5–11	3.54–24.06
*qMR.REG-4.3*	02428	JZ/B	76–76.27	5.3–10.82
*qMR.REG-4.4*	MR63	HN/NJ	106	7.71–9.1
	02428	NJ/SY/SZ	95.8–120.5	3.77–7.57
*qMR.REG-5.1*	MR63	SZ/B	5.5–10.19	4.25–6.03
*qMR.REG-5.4*	MR63	BJ/HN/JZ/NJ/SY/SZ/XZ/B	184.31–192.5	7.07–66.08
	02428	JZ/NJ	196.6–200.1	3.41–4.05
*qMR.REG-6.1*	02428	NJ/SY	10–24.5	3.36–5.48
*qMR.REG-6.2*	MR63	BJ/SY	41–41.5	4.91–9.94
	02428	JZ/SZ/B	49.78–49.8	6.62–12.1
*qMR.REG-6.3*	MR63	HN/JZ/NJ/SZ	84.9–106.8	4.19–17.47
*qMR.REG-6.4*	MR63	XZ/B	127.42	16.09–25.32
*qMR.REG-7.1*	MR63	NJ/SY/B	5.06–5.5	4.34–15.97
	02428	SZ/XZ	14–14.5	7.73–7.75
*qMR.REG-7.3*	MR63	BJ/HN/SZ/XZ	82.5–104.25	3.71–7.95
*qMR.REG-7.4*	02428	NJ/SY/B	116.5–118.62	4.69–6.27
*qMR.REG-8.1*	MR63	BJ/NJ	8.02–18	6.8–10.48
	02428	JZ/B	10–12.2	2.94–4.23
*qMR.REG-8.2*	MR63	HN/JZ/SY/XZ/B	34.4–59.14	3.47–44.07
	02428	SZ/XZ	42.8–57.39	3.81–7.76
*qMR.REG-8.3*	02428	NJ/SY	78.5–84.9	3.96–5.62
*qMR.REG-9.2*	MR63	BJ/NJ/SY/XZ/B	26.52–30.5	2.62–23.26
*qMR.REG-9.3*	02428	NJ/SZ/XZ/B	31.29–58	2.88–6.45
*qMR.REG-10.1*	MR63	BJ/SY/SZ	38.11–46.9	3.33–8.53
	02428	JZ/NJ/SY/SZ/XZ/B	20.06–39.5	3.16–11.67
*qMR.REG-10.2*	MR63	HN/JZ/NJ/XZ/B	86–95	7.19–45.49
*qMR.REG-11.1*	MR63	NJ/SZ	2.5–5	4.9–6.35
	02428	NJ/XZ	0–1	2.94–6.54
*qMR.REG-11.2*	MR63	BJ/JZ/XZ/B	31.45–35	3.11–22.05
*qMR.REG-11.3*	MR63	HN/SY	82.81–97.4	5.32–11.87
	02428	SY/SZ/B	90.77–99.2	5.38–6.73
*qMR.REG-12.1*	MR63	BJ/HN/NJ/SZ	11–26.61	5.67–11.4
*qMR.REG-12.2*	02428	XZ/B	42.45	12.38–12.93
*qMR.REG-12.3*	02428	SY/SZ	68–81.4	4.92–5.12
*qMR.REG-12.4*	MR63	SY/XZ/B	90.77–94.16	3.11–24.89
*qHR.REG-1.1*	MR63	BJ/HN/SY	4.5–24.7	3.89–532.0
*qHR.REG-1.2*	MR63	JZ/SZ/B	72.67–84.2	11.33–382.0
*qHR.REG-1.3*	MR63	NJ/XZ	161.4–165	23.7–501
	02428	JZ/NJ/SZ/XZ/B	161.9–168	5.28–12.2
*qHR.REG-2.1*	MR63	NJ/XZ	23.2	22.6–511
	02428	JZ/NJ	10.3–28.4	3.28–5.0
*qHR.REG-2.2*	MR63	BJ/HN/JZ/B	68.4–86.5	10.52–533.0
*qHR.REG-2.3*	02428	SZ/XZ/B	103.71–111	7.45–11.35
*qHR.REG-3.2*	MR63	HN/JZ/SZ	44.9–62.9	173.0–403.0
*qHR.REG-3.3*	02428	JZ/B	98.4–104	9.13–13.28
*qHR.REG-3.4*	MR63	BJ/NJ/SY/XZ/B	144.1–146.5	7.53–537.0
*qHR.REG-4.1*	MR63	BJ/HN/NJ/SY/XZ/B	5.06–34	17.53–535.0
*qHR.REG-4.2*	02428	JZ/SZ/B	63.5–65.59	3.9–6.48
*qHR.REG-4.3*	02428	NJ/XZ	86–88	4.1–6.53
*qHR.REG-5.1*	MR63	NJ/SY/XZ/B	14–15.8	20.61–382
*qHR.REG-5.2*	02428	JZ/NJ	130.1	2.69–9.25
*qHR.REG-5.3*	MR63	BJ/HN/XZ	184.7–192.53	213.0–534.0
	02428	SZ/XZ/B	196.99–199.54	4.8–7.1
*qHR.REG-6.1*	MR63	HN/JZ	15.3	177.0–252.0
	02428	NJ/SZ/XZ	24–39.06	3.83–8.96
*qHR.REG-6.2*	MR63	BJ/NJ/SY/XZ	40.99–52.3	9.19–541.0
*qHR.REG-6.3*	MR63	SZ/B	100.77–114.4	11.01–380.0
	02428	SY/B	125.68–140.08	5.28–5.52
*qHR.REG-7.1*	MR63	BJ/JZ/SY/SZ/XZ/B	5.28–36.4	6.31–533.0
	02428	JZ/SY/XZ/B	6.47–15	5.91–7.76
*qHR.REG-7.2*	MR63	HN/NJ	94–100	21.1–208
*qHR.REG-8.1*	MR63	BJ/HN/JZ/NJ/SY/SZ/XZ/B	22.5–58.7	16.32–539.0
	02428	NJ/SZ	48.2–52.95	2.99–7.95
*qHR.REG-9.1*	MR63	BJ/JZ/NJ/SY/SZ/XZ/B	29–36.1	6.12–530.0
*qHR.REG-9.2*	MR63	JZ/NJ/SY/SZ/XZ/B	57.5–87.58	3.88–14.4
*qHR.REG-10.2*	02428	NJ/SZ/XZ	17.42–39.9	3.65–9.84
*qHR.REG-10.3*	02428	SY/B	75.32–81.5	2.58–6.76
*qHR.REG-10.4*	MR63	HN/NJ/SY/XZ/B	92.5–93.8	14.36–516.0
*qHR.REG-11.1*	02428	SY/SZ	0.5–2	6.04–11.09
*qHR.REG-11.2*	MR63	BJ/HN/NJ/SY/XZ/B	27–31.9	10.57–537.0
*qHR.REG-11.3*	MR63	JZ/SZ	58.35–65.5	163.0–383.0
	02428	JZ/NJ/B	49.49–58.3	3.19–6.5
*qHR.REG-12.1*	02428	NJ/SZ/B	2.28–24	2.67–11.15
*qHR.REG-12.2*	02428	JZ/XZ	42.4–49.5	4.65–7.81
*qHR.REG-12.3*	MR63	BJ/NJ/SY/SZ/XZ/B	82–94.2	15.5–535.0

*GL* grain length, *GW* grain width, *BR* brown rice rate, *MR* milled rice rate, *HR* head rice rate

^a^Population

^b^Environment

^c^Position

B: Best linear unbiased prediction (BLUP)

### QTL detected by CSL

A total of 36, 24, 21, 19, and 24 QTLs were detected by CSL for GL, GW, BR, MR and HR, respectively, whose information was listed in Table S4. Positions, LOD scores, PVE, and additive effect of stable QTLs were given in Table 6. For GL, 22 QTLs were stable, 8 of which were BI loci, i.e. *qGL.CSL-1.1*, *qGL.CSL-2.1*, *qGL.CSL-2.2*, *qGL.CSL-3.4*, *qGL.CSL-3.5*, *qGL.CSL-3.6*, *qGL.CSL-4.2* and *qGL.CSL-10.2*. For GW, 13 QTLs were stable, 2 of which were BI loci, i.e. *qGW.CSL-1.2* and *qGW.CSL-5.1*. For BR, MR, and HR, there were 8, 7, and 6 QTLs were stable, but no BI locus was found. Most stable QTLs also had the same directions of additive effect across populations and environments.

**Table 6:** Stable QTLs detected by CSL

**Loci**	**Pop **^**a**^	**Env.**^**b**^	**Pos. (cM)**^**c**^	**LOD**	**PVE (%)**^**d**^	**Add.**^**e**^
*qGL.CSL-1.1*	MR63	SY/SZ/XZ	14.21–15.93	8.56–9.47	0.35–6.10	-0.22 to -0.16
	02428	JZ/M	11.78–22.84	3.03–4.42	3.55–6.16	-0.24 to -0.22
*qGL.CSL-1.2*	MR63	SY/SZ	53.07–60.30	3.84–4.72	1.93–2.32	-0.10 to -0.09
*qGL.CSL-1.3*	MR63	HN/SZ/M	111.77	3.33–11.14	1.31–10.70	0.07 to 0.15
*qGL.CSL-1.4*	MR63	BJ/HN/JZ/NJ/SZ/XZ/M	138.05–138.48	5.80–19.02	3.93–8.03	-0.25 to -0.10
*qGL.CSL-2.1*	MR63	BJ/SY/SZ/XZ	10.81–22.33	2.86–10.87	0.12–7.13	0.10 to 0.17
	02428	JZ/NJ	2.04–18.78	4.66–6.41	3.76–7.87	0.15 to 0.26
*qGL.CSL-2.2*	MR63	XZ/M	54.49–58.46	17.17–20.75	0.95–5.77	-1.35 to 0.19
	02428	NJ/SY	70.06	2.96–3.05	2.35–3.94	-0.10
*qGL.CSL-2.3*	MR63	JZ/SZ/M	112.08–129.61	6.16–12.50	1.85–7.48	-0.18 to 0.28
*qGL.CSL-3.1*	MR63	HN.NJ/SY/SZ/XZ	2.22–14.87	3.32–11.99	0.49–5.14	0.19 to 0.40
*qGL.CSL-3.4*	MR63	BJ/HN/JZ/NJ/SY/SZ/XZ/M	91.86–101.41	15.57–39.38	0.93–23.73	0.29 to 0.52
	02428	JZ/NJ/SZ/M	91.43–93.69	5.13–25.20	7.56–26.25	0.18 to 0.46
*qGL.CSL-3.5*	MR63	JZ/XZ/M	113.88–114.90	12.34–13.65	0.59–7.75	0.20 to 0.31
	02428	SY/SZ/XZ/M	114.90	6.94–10.56	10.25–18.33	0.21 to 0.31
*qGL.CSL-3.6*	MR63	SY/SZ	139.32–148.85	6.44–14.10	3.88–6.40	0.16 to 0.24
	02428	JZ/NJ/SY/SZ/M	136.65–149.60	3.49–11.77	5.04–15.52	0.16 to 0.36
*qGL.CSL-4.2*	MR63	HN/NJ/M	95.75–98.17	4.93–11.83	1.19–7.01	0.19 to 0.24
	02428	JZ/SY/SZ/XZ/M	85.99–99.75	4.33–8.74	5.59–14.90	0.16 to 0.25
*qGL.CSL-5.2*	02428	SZ/XZ/M	173.73	3.89–5.48	5.48–8.27	0.14 to 0.20
*qGL.CSL-7.1*	MR63	NJ/SY/XZ	29.19–41.82	13.40–114.17	9.03–21.52	0.24 to 1.35
*qGL.CSL-7.2*	MR63	JZ/NJ/SZ/XZ/M	67.18–68.15	4.55–47.61	0.66–22.02	0.16 to 0.33
*qGL.CSL-7.3*	MR63	NJ/M	99.96–104.25	4.44–22.85	1.27–6.69	0.11 to 0.50
*qGL.CSL-8.1*	MR63	BJ/HN/NJ/JZ/XZ	16.71–21.95	4.24–9.65	0.22–5.77	0.15 to 0.29
*qGL.CSL-8.2*	MR63	BJ/NJ/SZ/M	62.47–80.30	4.09–15.24	0.97–14.75	-0.18 to -0.08
*qGL.CSL-10.1*	MR63	JZ/SY/SZ/XZ	36.54–46.21	3.64–6.06	0.24–3.36	-0.23 to -0.12
*qGL.CSL-10.2*	MR63	JZ/SY/SZ/XZ	67.00–73.05	4.88–18.10	0.19–11.78	0.16 to 0.35
	02428	NJ/SY/SZ/XZ/M	73.05	5.89–8.52	7.14–10.77	0.19 to 0.24
*qGL.CSL-11.1*	MR63	BJ/HN/NJ/SZ/XZ/M	6.89–21.55	10.23–126.38	5.56–23.57	0.12 to 1.43
*qGW.CSL-1.2*	MR63	HN/JZ/NJ/SY/XZ/M	42.13–60.57	4.99–23.76	2.37–20.68	0.03 to 0.12
	02428	SZ/M	72.67	2.74–6.08	2.63–6.28	-0.09 to -0.06
*qGW.CSL-1.4*	MR63	JZ/NJ/XZ/M	161.42–163.91	5.35–33.51	2.46–22.07	-0.14 to -0.03
*qGW.CSL-3.1*	02428	NJ/XZ/M	30.40	3.41–6.61	5.36–8.89	-0.09 to -0.06
*qGW.CSL-3.3*	MR63	HN/SY/SZ/M	149.60–165.16	4.24–21.87	1.42–7.68	-0.15 to -0.05
*qGW.CSL-4.1*	MR63	JZ/SY/M	39.89–40.90	3.02–13.85	2.81–5.08	-0.06 to -0.04
*qGW.CSL-4.3*	02428	SY/SZ/M	108.78	5.21–9.77	5.58–12.30	-0.08 to -0.05
*qGW.CSL-5.1*	MR63	BJ/HN/JZ/NJ/SY/SZ/XZ/M	27.48–32.77	9.33–94.36	6.36–47.51	-0.29 to -0.08
	02428	JZ/NJ/SY/SZ/XZ/M	28.68	9.92–27.18	18.98–37.53	-0.15 to -0.11
*qGW.CSL-5.2*	MR63	BJ/HN/SZ	131.89–144.19	3.14–3.92	0.57–4.75	-0.09 to -0.04
*qGW.CSL-5.3*	MR63	BJ/HN/SY/SZ/M	177.26–184.75	4.95–16.76	3.32–6.93	0.04 to 0.07
*qGW.CSL-7*	MR63	NJ/XZ	95.10–99.96	4.22–18.18	1.97–9.36	-0.12 to -0.05
*qGW.CSL-8*	MR63	JZ/NJ/SY/M	18.25–21.95	5.69–27.00	4.23–10.06	-0.13 to -0.07
*qGW.CSL-12.1*	MR63	HN/NJ/SY/XZ/M	0.22–13.04	3.35–9.46	1.57–5.27	-0.08 to -0.03
*qGW.CSL-12.3*	02428	JZ/NJ/SY/SZ/XZ/M	81.39–89.21	6.95–10.82	9.92–17.30	-0.08 to -0.06
*qBR..CSL-2.2*	02428	XZ/M	70.06–80.74	2.83–3.68	5.44–6.82	0.42 to 0.52
*qBR..CSL-2.4*	MR63	HN/M	146.10–148.52	3.09–7.38	2.63–12.20	0.41 to 1.91
*qBR..CSL-3*	MR63	HN/NJ/M	4.84–22.94	3.26–5.14	4.51–8.96	1.11 to 2.14
*qBR..CSL-5.1*	MR63	HN/M	29.73–30.82	2.63–5.99	3.76–5.34	-1.15 to -0.72
*qBR..CSL-5.2*	MR63	SZ/XZ/M	184.31–192.53	5.41–15.4	9.98–16.19	0.93 to 2.30
*qBR..CSL-6*	MR63	SY/M	117.80–132.04	3.14–8.61	6.59–7.86	0.89 to 1.10
*qBR..CSL-7.2*	MR63	XZ/M	68.15–82.40	3.66–3.67	3.18–5.64	-0.98 to -0.49
*qBR..CSL-9*	02428	NJ/M	57.50–67.61	4.78–6.94	6.19–9.13	-0.67 to -0.49
*qMR.CSL-1.1*	MR63	SZ/XZ/M	14.21–25.33	2.57–5.72	1.74–10.01	0.41 to 2.52
*qMR.CSL-1.2*	MR63	HN/M	130.85–140.51	4.08–4.74	3.22–6.01	0.46 to 1.04
*qMR.CSL-2.1*	02428	XZ/M	80.74	4.45–5.10	7.34–9.10	0.60 to 0.97
*qMR.CSL-2.3*	MR63	HN/M	148.52	6.83–7.08	4.76–10.63	0.63 to 1.47
*qMR.CSL-3.2*	02428	XZ/M	113.00–114.90	3.99–4.53	6.91–7.37	0.74 to 1.23
*qMR.CSL-5.2*	MR63	BJ/HN/NJ/SY/SZ/XZ/M	180.55–194.38	3.02–22.65	1.87–19.02	0.69 to 1.64
*qMR.CSL-12*	MR63	BJ/XZ	26.61–33.40	2.56–4.40	3.67–5.26	-1.13 to 2.35
*qHR.CSL-2.2*	MR63	XZ/M	109.41–113.66	3.88–9.28	3.43–9.40	-5.06 to -1.59
*qHR.CSL-3.3*	MR63	SZ/XZ/M	91.86–98.38	3.84–5.70	4.02–7.24	-4.98 to -2.20
*qHR.CSL-5.1*	MR63	NJ/SY/M	10.19–14.19	7.21–9.81	6.58–19.22	1.63 to 4.14
*qHR.CSL-6.2*	MR63	XZ/M	98.73–114.37	3.78–7.82	3.33–7.79	-3.64 to -1.21
*qHR.CSL-10.2*	MR63	BJ/XZ/M	75.84–93.84	3.06–26.05	2.91–28.90	3.23 to 5.14
*qHR.CSL-12.1*	MR63	XZ/M	5.01–10.09	3.64–7.72	3.39–7.09	2.02 to 2.73

*GL* grain length, *GW* grain width, *BR* brown rice rate, *MR* milled rice rate, *HR* head rice rate

^a^Population

^b^Environment

^c^Position

^d^Percentage of phenotypic variance explained

^e^Additive effect

M: Mean values across environments

### QTL detected by MET

**Fig. 2:**
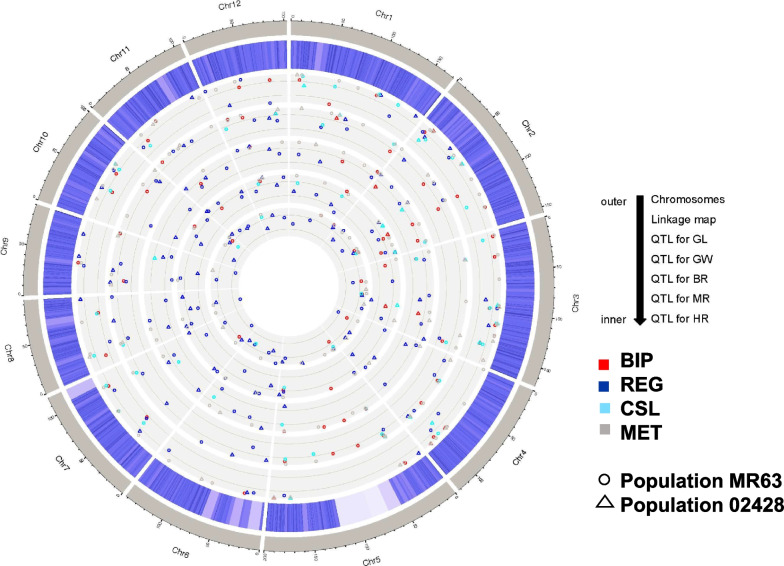
Stable QTLs for grain length (GL), grain width (GW), brown rice rate (BR), milled rice rate (MR), and head rice rate (HR) along the whole genome

A total of 38, 29, 25, 18, and 22 QTLs were detected by MET for GL, GW, BR, MR and HR, respectively, whose information was listed in Table S5. Since these QTLs were detected by QTL by environment interaction analysis instead of by single-environmental analysis, it is not suitable to determine if they were stably detected or not. To simplify the comparison of mapping results with the other two methods, we also regarded these QTLs as stable QTLs in this study. For GL, 14 QTLs were BI loci, i.e. *qGL.MET-1.1*, *qGL.MET-2.1*, *qGL.MET-2.2*, *qGL.MET-3.4*, *qGL.MET-3.5*, *qGL.MET-3.6*, *qGL.MET-4.3*, *qGL.MET-4.4*, *qGLMET-5.1*, *qGL.MET-6.1*, *qGL.MET-8.1*, *qGL.MET-10.1*, *qGL.MET-10.2*, and *qGL.MET-11.2*. For GW, eight BI loci were detected, i.e. *qGW.MET-1.2*, *qGW.MET-2.2*, *qGWMET-3.3*, *qGW.MET-5.1*, *qGW.MET-8*, *qGW.MET-9.1*, *qGW.MET-9.2*, and *qGW.MET-11.1*. For BR, one BI locus was detected, i.e. *qBR.MET-3.2*. For MR, one BI locus was detected, i.e. *qMR.MET-12*. For HR, two BI loci were detected, i.e. *qHR.MET-2.3* and *qHR.MET-3.2*. Positions of stable QTLs along the whole genome were shown in Fig. 2.

### Common loci among different QTL mapping methods

**Fig. 3:**
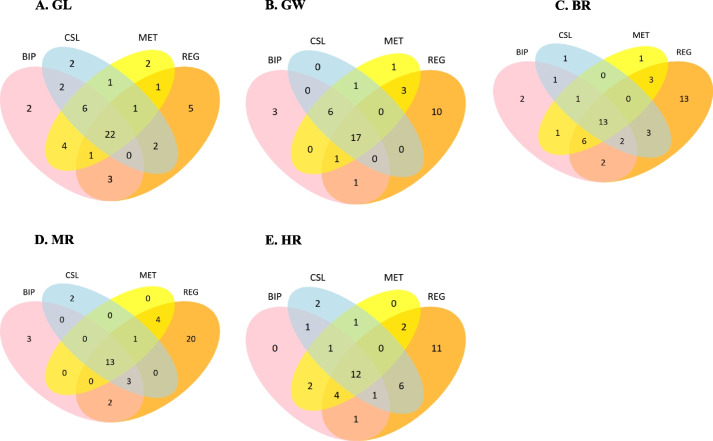
Common QTLs among different QTL mapping methods

All detected QTLs were used for comparison among different QTL mapping methods. The number of common loci from BIP, REG, CSL, and MET were shown in Fig. 3 for all the five traits. For GL, BIP, REG, CSL, and MET detected 40, 35, 29 and 28 QTLs, respectively, of which 22 QTLs were common for the four methods. For GW, REG, BIP, CSL, and MET detected 28, 32, 25 and 29 QTLs, respectively, of which 16 QTLs were common for the four methods. For BR, BIP, REG, CSL, and MET detected 28, 42, 21 and 25 QTLs, respectively, of which 13 QTLs were common for the four methods. For MR, BIP, REG, CSL, and MET detected 21, 43, 19 and 18 QTLs, respectively, of which 13 QTLs were common for the four methods. For HR, BIP, REG, CSL, and MET detected 22, 37, 23 and 22 QTLs, respectively, among which 11 QTLs were common for the four methods. More than half of the QTLs from BIP, REG, CSL, and MET were detected by at least two mapping methods, which reflected the reliability of these QTLs. The ratios of common loci (number of common loci / QTLs detected by certain method) were more than 75% by MET and CSL methods for GL, while these values decreased to 50% for HR by BIP and MET. REG detected relatively more QTLs than the other methods.

### Common region harboring QTLs for different traits

**Fig. 4:**
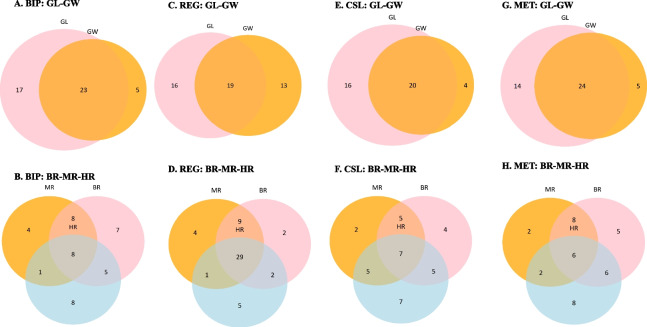
Common QTLs between grain shape traits and among milling quality traits

Considering the trait correlations between GL and GW, and among BR, MR and HR, comparison of QTLs was conducted in two groups of traits separately, i.e. GS and MQ traits. Common regions harboring QTLs for GS and MQ traits were shown in Fig. 4 for the three mapping methods separately. For GL and GW, there were 23, 19, 21 and 24 common QTLs from BIP, REG, CSL and MET, respectively. Generally, directions of additive effects of these common QTLs were opposite on the two traits. For example, *qGL.BIP-3.2* and *qGW.BIP-3.1* located at 30–34 cM and at 30.5 cM on chromosome 3, respectively. Additive effect of qGL.BIP-3.2 from 0.18 to 0.29 cm, while that of *qGW.BIP-3.1* ranged from -0.07 to -0.04 cm. This opposite effect was consistent with the negative trait correlation between GL and GW. Similar consistencies were found for *qGL.BIP-4.5* and *qGW.BIP-4.3*, *qGL.BIP-5.1* and *qGW.BIP-5.1*, *qGL.CSL-3.7* and *qGW.CSL-3.3*, etc. Some exceptional cases were also observed. For example, *qGL.CSL-5.2* and *qGW.CSL-5.3* at 173.73 cM and at 176.81–184.31 cM on chromosome 5, contributed additive effect from 0.14 to 0.20 and 0.04 to 0.07, respectively (Tables S2 and S4). For BR, MR and HR, there were 8, 29, 7 and 6 common QTLs from BIP, REG, CSL and MET, respectively. Although significant positive correlations were observed between the three traits, there were only several common QTLs exhibiting same effect direction of additive for all the three traits at the same time. *qBR.CSL-1.1*, *qMR.CSL-1.1* and *qHR.CSL-1.1* were located closely, and additive effects were all positive. Similarly, *qBR.CSL-3*, *qMR.CSL-3.1* and *qHR.CSL-3.1*; *qBR.CSL-5.2*, *qMR.CSL-5.2* and *qHR.CSL-5.3*. *qBR.CSL-2.3*, *qMR.CSL-2.2* and *qHR.CSL-2.2* were located closely, and the additive effects of them were all negative (Table S4).

**Fig. 5:**
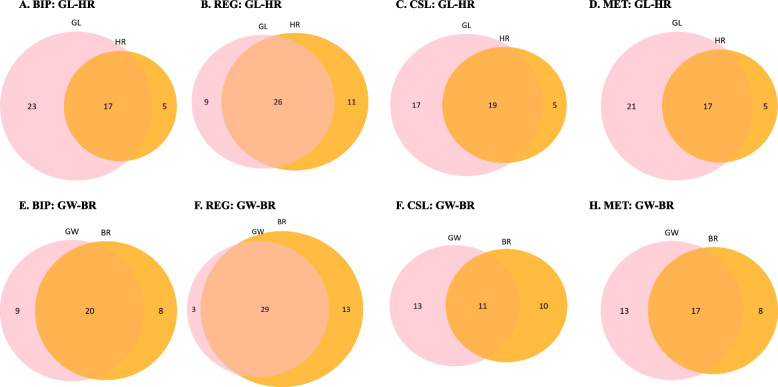
Common QTLs between grain length (GL) and head rice rate (HR), and between grain width (GW) and brown rice rate (BR)

As we mentioned, the correlation between GL and HR was also significant, and so was the correlation between GW and BR. Common QTLs between GL and HR and those between GW and BR are shown in Fig. 5. There were 17, 26, 19 and 17 common QTLs between GL and HR from BIP, REG, CSL and MET, respectively. And there were 20, 29, 11 and 17 common QTLs between GW and BR from BIP, REG, CSL and MET, respectively. It can be concluded that higher correlation between traits leaded to more common QTLs for different traits, no matter which mapping method was used. Common QTLs were not only observed between traits belong to the same group, but also between traits from different groups.

### Design of the target genotype based on the identified QTLs

Nineteen QTLs for GL and HR were detected by all the four methods and had consistent directions of additive effect across different environments. Eleven of them affected both GL and HR, five of which increased or decreased both traits, and the other five had different directions of effects for the two traits. For QTLs only affecting GL, the alleles increasing GL were set as the favorable one; for QTLs only affecting HR, the alleles increasing HR were set as the favorable one; for QTLs affecting both GL and HR, the alleles increasing HR were set as the favorable one. The target genotype at the 19 QTLs is given in Table 7. Four individuals from the two populations were picked up as parents in breeding design, i.e. MH63-ILs-166, 02428-ILs-185, MH63-ILs-65 and MH63-ILs-168, which had only 4, 5, 5 and 5 different QTL genotypes compared with the target genotype. Their genotypes were also given in Table 7. The predicted values of the selected lines with these QTLs under certain environments were also provided (Table S6).

**Table 7:** Nineteen QTLs detected by all the four methods and had consistent additive effect directions among different environments

qGL.BIP^a^	qHR.BIP^b^	Position (cM)^c^	Distance (cM)^d^	MR63-166	02428–185	MR63-65	MR63-168	Target genotype
1.1	NA^e^	11.5	0	A^f^	B	A	A	B
1.3	1.1	109.2	0.6	B	B	A	B	B
2.1	NA	12.6	0.4	A	A	A	A	A
3.1	NA	6.5	0.6	A	B	A	A	A
3.3	3.1	60	0.4	A	B	B	A	B
3.4	3.2	94.6	0.3	B	A	B	B	B
3.6	3.3	137.6	0.1	B	A	A	B	B
4.4	4.3	85.4	0.3	A	B	A	A	A
4.5	NA	107	0.1	A	B	A	A	A
5.1	5.1	24.7	0.1	A	A	A	A	A
NA	5.3	134	0.2	A	A	A	A	A
5.5	NA	194	0.1	A	A	A	A	A
6.2	6	81	0.1	A	B	B	A	B
7.3	7.2	101	0.1	B	B	A	A	B
9	NA	38	1.9	A	A	A	A	A
10.2	10.1	68.9	0.1	A	B	A	A	B
11.1	11	15.4	0.1	A	A	A	A	A
NA	12	9	0.2	A	A	A	A	A
12.1	NA	41.5	0.3	A	A	A	A	A

^a^QTL name for GL detected by BIP

^b^QTL name for HR detected by BIP

^c^For simplicity, it is average position of GL QTL across all the environments which detected this QTL. If this QTL doesn’t affect GL, it is the average position of HR QTL

^d^Distance between QTL and the nearest marker

^e^NA means no QTL was detected for the trait at this position

^f^Genotype A meant the allele from parent MR63, and genotype B meant the allele from parent 02428

For the four selected individuals, we performed two single crosses, six top crosses and six double crosses to generate the target genotype, respectively, by using Blib. These crosses and the corresponding percentage of the designed genotype in the DH population were also provided in Table 8. The percentage of the designed genotype for SC1 was 0.0014, higher than that for SC2. Percentage for TC2 was 0.0022, which was the highest among the six top crosses. Percentage for DC2 was 0.0018, which was the highest among the six double crosses. TC2 and DC2 had the common three parents, but different cross design led to different genetic structures in the two populations, as well as different percentages of the designed genotype.

**Table 8:** Percentage of the designed genotype in doubled haploid population derived from a single cross, top cross or double cross

**Single cross (SC), Top cross (TC) or Double cross (DC)**	**Mean**	**Standard error**
SC1: MR63-166/02428–185	0.0014	0.0012
SC2: MR63-168/02428–185	0.0007	0.0008
TC1: MR63-65/02428–185//MR63-166	0.0011	0.0011
TC2: MR63-65/MR63-166//02428–185	0.0022	0.0015
TC3: MR63-166/02428–185//MR63-65	0.0008	0.0009
TC4: MR63-168/02428–185//MR63-166	0.0004	0.0006
TC5: MR63-168/MR63-166//02428–185	0.0011	0.0010
TC6: MR63-166/02428–185//MR63-168	0.0002	0.0005
DC1: MR63-65/MR63-166//02428–185/MR63-166	0.0014	0.0012
DC2: MR63-65/02428–185//MR63-166/02428–185	0.0018	0.0014
DC3: MR63-166/MR63-65//02428–185/MR63-65	0.0007	0.0008
DC4: MR63-168/MR63-166//02428–185/MR63-166	0.0004	0.0006
DC5: MR63-168/02428–185//MR63-166/02428–185	0.0014	0.0012
DC6: MR63-166/MR63-168//02428–185/MR63-168	0.0002	0.0005

## Discussion

### More reasonable mapping results based on linkage map

In previous mapping work with BC_2_RIL [23, 24], we adopted physical map rather than linkage map for QTL identification. However, according to the underlying principles in methodology for popular QTL mapping packages, such as IciMapping [25], linkage map would be preferred. We compared our mapping results based on new map with same mapping method of BIP (Table S2) to our previous report [24]. It’s notable that some key loci, e.g. *GL-3* is detected in more locations with larger LOD values. Despite this, for all the other grain shape loci, more stable detection with higher significance were found. For example, the mean LOD value was 12.3 (Table S2) now vs 9.1 in previous report [24] for GL. The same is true (11.7 vs 10.4) for GW. This set of BC_2_RIL lines with maps and seeds are now publicly available, which may provide useful materials and information for more effective genetic dissection of complex traits. This population would be open and accessible through following URL (https://rfgb.rmbreeding.cn/download/publicDataDownload) provided by the Functional Genomic Breeding (FGB) platform [34]. This kind of population has previously already proven its value in genetic analyses for appearance quality [24] and may be useful for other quality traits, e.g. mineral concentration [35].

### Comparison of QTLs detected in the two populations

As shown in Tables S2, S3, S4, and S5, there were some QTLs detected in both populations for the five traits by all the three QTL mapping methods. Stable QTLs for the two populations could be observed in Fig. 2. For QTLs detected by BIP and CSL, reliability of these QTLs was higher than those stable in only one population, and followed by the unstable QTLs. As we presented before, directions of additive effects of these QTLs were mostly consistent for the two populations. However, values of additive effects varied significantly across populations and environments. Generally, if a QTL for the two populations was detected in the same environment, similar or larger effects was observed for MH63-ILs than that for 02428-ILs. For example, *qGL.BIP-10.2* was detected in six environments for MH63-ILs and five environments for 02428-ILs (Table 4 and S2). In environments SY, SZ, XZ, and BLUP, *qGL.BIP-10.2* was detected for both populations. Its additive effect was larger in MH63-ILs than that in 02428-ILs except in SZ. The larger QTL effects may be related to the stricter selection on MH63-ILs. Further study is needed to prove this inference.

### QTLs regions with multiple effects

As we mentioned above, some common QTLs were detected within and also between GS and MQ trait groups. As an example, a total of nine regions harboring these QTLs are located within an interval shorter than 20 cM (Table S7). Of these regions, three were located on chromosome 5, with an average size of 17.9 cM corresponding to about 1.6 Mb; two on chromosome 3 with an average size of 3.8 cM (2.4 Mb); other four with averaged size of 4.2 cM (0.5 Mb) on chromosomes 1, 2, 10, and 11 respectively. Especially, *qGL.BIP-3.4* which contains both the major effect QTL for grain length, *GS3* [36] and *qHR.BIP-3.2* for head rice rate. It is evident that slender grains tend to be broken more easily during milling processing [2]. It’s highly possible that QTLs governing rice grain size may be tightly linked to, or pleiotropic loci for HR. These regions may be the causal factors underlying MQ-GS trait correlations / genetic overlaps. Additional work is in needed for further identification.

### Comparison of detected QTLs with previous studies

A total of 246 QTLs for the five traits reported in 20 previous studies were collected for comparison with results of this study, of which 63, 67, 32, 39 and 44 QTLs affected GL, GW, BR, MR and HR, respectively (Table S2). For GL, a total of 60 reported QTLs in previous studies were detected in this study, of which 47, 42, 36, and 43 were identified by BIP, REG, CSL and MET, respectively. Twenty reported GL QTLs were detected by all the four mapping methods. For GW, 54 QTLs reported QTLs were detected, of which 32, 40, 27, and 34 were identified by BIP, REG, CSL and MET, respectively. Eighteen reported GW QTLs were detected by all the four mapping methods. For BR, 33 reported QTLs were detected, among which 16, 27, 11, and 9 were identified by BIP, REG, CSL and MET, respectively. Three reported BR QTLs were detected by all the four mapping methods. For MR, 31 reported QTLs were detected, of which 13, 29, 14, and 9 were identified by BIP, REG, CSL and MET, respectively. Five reported MR QTLs were detected by all four mapping methods. For HR, 36 reported QTLs were detected, of which 16, 29, 16, and 16 were identified by BIP, REG, CSL and MET, respectively. Six reported HR QTLs were detected by all four mapping methods. Comparison results showed the reliability of detected QTLs and the relative consistency of the four mapping methods in this study.

There were eight novel QTLs detected by this study that were not reported in previous studies, and were stable in all the four mapping methods, i.e. *qGL.BIP-5.4*, *qGL.BIP-8.2*, *qGL.BIP-10.1*, *qGW.BIP-1.4*, *qGW.BIP-3.1*, *qGW.BIP-12.3*, *qMR.BIP-2.1* and *qHR.BIP-2.1*. These QTLs may provide useful information in the future breeding of rice.

### Application of detected QTLs in rice breeding

A large amount of QTL mapping studies have been conducted for various traits in rice in the last two decades, and how to utilize so many detected QTLs or genes becomes a challenge to the breeders. In this study, the simulation platform Blib has been adopted to design the target genotypes using identified QTLs. Two significantly related traits, GL and HR, were selected as examples to demonstrate the design process, which was quite important in plant breeding. According to the simulation results, the target genotype with larger HR and relatively longer GL could be obtained when 1000 DHs were generated from the cross between or among the selected individuals in the two mapping populations, no matter single cross, top cross, or double cross was adopted.

The two mapping populations in this study are generated by consecutive backcrossing, which are close to CSSL populations in two directions, the MH63-ILs using MH63 as the recurrent parent, and the 02428-ILs using 02428 as the recurrent parent. The individuals of MH63-ILs had higher genotypic similarity with the parent MH63, of which 91.30% were same as parent MH63, 7.08% were same as parent 02428, and 1.62% were missing. Genotypes of individuals in 02428-ILs were closer to parent 02428, among which 19.88% was same as parent MH63, 76.55% was the same as parent 02428, and 3.57% was missing. But more abundant genetic variations can be found in the two populations than the two parents. When the target genotype is defined in simulation study, it is easy to select suitable parents for crossing, which are in genotype at limited number of QTL. In addition, these parents are complementary, i.e. at least one of the parents was homogenous with target genotype. The development of mapping populations and simulation tools and procedures for the target genotype provides a potential way of utilizing of the detected QTLs.

## Conclusions

A set of reciprocal introgression lines with a genetic map was provided for genetic dissection of complex traits. The procedure of selecting more suitable analytical methods for this kind of population were also presented by using MQ-GS trait correlations / genetic overlaps as example. Besides eight new loci, nine loci-harboring regions for different traits and background independent loci were reported. Background independent (BI) loci were also found for each MQ and GS traits. All these information together with the simulation on breeding design may provide useful guidelines for rice molecular breeding.

### Supplementary Information

**Additional file 1: Table S1.** Summary statistics of the five traits for the two populations in different environments. **Table S2.** Detail information of all QTLs detected by BIP. **Table S3.** Detail information of all QTLs detected by REG. **Table S4.** Detail information of all QTLs detected by CSL. **Table S5.** Detail information of all QTLs detected by MET. **Table S6.** Predicted genotypic values of the selected lines and target genotype in each population and environment. Additive effect of each QTL for calculating the genotypic values was picked up from the BIP mapping results at the nearest position of the average QTL position across in Table 7. **Table S7.** Nine regions harboring QTLs for MQ-GS traits.

## Data Availability

Data for the 424 BC_2_RIL lines including the genotypic data, maps and seed availability are provided through the following URL https://rfgb.rmbreeding.cn/download/publicDataDownload. Seeds can be requested through the following URL https://rfgb.rmbreeding.cn/search/germplasm/seedrequest or email to the corresponding authors.

## Abbreviations

MQ: Milling quality
GS: Grain shape
RIL: Recombinant inbred line
GL: Grain length
GW: Grain width
FGB: Functional Genomic Breeding
BR: Brown rice rate
MR: Milled rice rate
HR: Head rice rate
QTL: Quantitative trait locus
